# Enzootic Angiostrongyliasis in Shenzhen, China

**DOI:** 10.3201/eid1412.080695

**Published:** 2008-12

**Authors:** Ren-Li Zhang, Mu-Xin Chen, Shi-Tong Gao, Yi-Jie Geng, Da-Na Huang, Jian-Ping Liu, Yuan-Liang Wu, Xing-Quan Zhu

**Affiliations:** Shenzhen Center for Disease Control and Prevention, Shenzhen, People’s Republic of China (R.-L. Zhang, M.-X. Chen, S.-T. Gao, Y.-J. Geng, D.-N. Huang, J.-P. Liu, Y.-L. Wu); South China Agricultural University, Guangzhou, People’s Republic of China (M.-X. Chen, X.-Q. Zhu)

**Keywords:** Angiostrongylus cantonensis, eosinopilic meningitis, Shenzhen, human, rates, snails, letter

**To the Editor:** *Angiostrongylus cantonensis* is a zoonotic parasite that causes eosinophilic meningitis in humans after they ingest infective larvae in freshwater and terrestrial snails and slugs, paratenic hosts (such as freshwater fish, shrimps, frogs, and crabs), or contaminated vegetables. With the increase of income and living standards, and the pursuit of exotic and delicate foods, populations around the world have seen angiostrongyliasis become an important foodborne parasitic zoonosis (*1*–*9*).

Shenzhen municipality is situated in the most southern part of mainland People’s Republic of China between the northern latitudes of 22°27′ to 22°52′ and eastern longitudes of 113°46′ to 114°37′; it shares a border with the Hong Kong Special Administrative Region, China, in the south. The climate is subtropical, with an average annual temperature of 23.7°C. The city is 1,952.84 km^2^ and has a population of 10 million.

Since 2006, thirty-two sporadic cases of human eosinophilic meningitis caused by consumption of undercooked aquacultured snails have been documented in Shenzhen (Shenzhen Center for Disease Control and Prevention, unpub. data). To identify the source of these infections and assess the risk for an outbreak of eosinophilic meningitis, we conducted a survey to investigate whether *A. cantonensis* occurs in wild rats and snails in Shenzhen.

To examine *A. cantonensis* infection in intermediate host snails, 302 terrestrial snails (*Achatina fulica*) were collected from 10 investigation sites across Shenzhen, and 314 freshwater snails (*Pomacea canaliculata*)were sampled from 6 investigation sites. We examined the snails for *A. cantonensis* larvae by using pepsin digestion standardized procedures (*3*). To survey the prevalence of adult *A. cantonensis* in definitive host rats, we collected 187 *Rattus norvegicus* rats and 121 *R. flavipectus* rats collected from 4 sites where positive snails positive for *A. cantonensis* were found. These rats were examined for the presence of adult *A. cantonensis* in their cardiopulmonary systems.

*A. cantonensis* larvae were found in 96 (15.6%) of 616 examined snails. Of these, *P. canaliculata* had an average infection rate of 20.7% (65/314), significantly higher (p<0.01) than that of *A. fulica* (10.3%, 31/302), an indication that *P. canaliculata* may be the principal intermediate host for *A. cantonensis* in Shenzhen. *A. cantonensis* adults were recovered from the cardiopulmonary systems of 37 (12%) of 308 examined rats. Infection rate for *R. norvegicus* rats was 16.6% (31/187), significantly higher (p<0.01) than that for *R. flavipectus* (4.9%, 6/121), an indication that *R. norvegicus* may be the principal definitive host for *A. cantonensis* in Shenzhen, possibly due to the rat’s preference for eating snails. Infection rates were higher for female rats (25.6% for *R. norvegicus* and 7.8% for *R. flavipectus*) than for male rats (8.9% for *R. norvegicus,* 2.9% for *R. flavipectus*), possibly because female rats eat more snails to supply proteins for reproduction. This report of enzootic *A. cantonensis* infection in wild rats and snails in Shenzhen demonstrates the existence of natural origins of infection with *A. cantonensis* for humans in this city.

Persons in Shenzhen eat raw or undercooked freshwater and terrestrial snails and slugs. This practice provides opportunities for infection with *A. cantonensis,* particularly given that *P. canaliculata* has been aquacultured intensively for human consumption. The prevalence of *A. cantonensis* in wild rats and snails in Shenzhen poses substantial risk for future outbreaks of human eosinophilic meningitis. Moreover, public health officials, epidemiologists, researchers, clinical technicians, medical practitioners, parasitologists, and veterinarians, as well as the general public, should be aware of such risks, and integrated strategies should be taken to reduce or eliminate such risks.
